# The Cambridge Centre for Ageing and Neuroscience (Cam-CAN) study protocol: a cross-sectional, lifespan, multidisciplinary examination of healthy cognitive ageing

**DOI:** 10.1186/s12883-014-0204-1

**Published:** 2014-10-14

**Authors:** Meredith A Shafto, Lorraine K Tyler, Marie Dixon, Jason R Taylor, James B Rowe, Rhodri Cusack, Andrew J Calder, William D Marslen-Wilson, John Duncan, Tim Dalgleish, Richard N Henson, Carol Brayne, Cam-CAN, Fiona E Matthews

**Affiliations:** Department of Psychology, University of Cambridge, Cambridge, CB2 3EB UK; School of Psychological Sciences, The University of Manchester, Brunswick Street, Manchester, M13 9PL UK; MRC Cognition and Brain Sciences Unit, 15 Chaucer Road, Cambridge, CB2 7EF UK; Department of Clinical Neurosciences, University of Cambridge, Cambridge, UK; Behavioural and Clinical Neuroscience Institute, Cambridge, UK; Brain and Mind Institute, University of Western Ontario, London, Ontario N6A 5B7 Canada; Department of Experimental Psychology, University of Oxford, Oxford, UK; Department of Public Health and Primary Care, Institute of Public Health, University of Cambridge, Cambridge, UK; MRC Biostatistics Unit, Institute of Public Health, Cambridge Biomedical Campus, Cambridge, CB2 0SR UK

**Keywords:** Healthy ageing, Brain ageing, Brain imaging, Epidemiology, Cognition, Magnetoencephalography, Functional MRI, Structural MRI, Brain networks, Lifespan

## Abstract

**Background:**

As greater numbers of us are living longer, it is increasingly important to understand how we can age healthily. Although old age is often stereotyped as a time of declining mental abilities and inflexibility, cognitive neuroscience reveals that older adults use neural and cognitive resources flexibly, recruiting novel neural regions and cognitive processes when necessary. Our aim in this project is to understand how age-related changes to neural structure and function interact to support cognitive abilities across the lifespan.

**Methods/Design:**

We are recruiting a population-based cohort of 3000 adults aged 18 and over into Stage 1 of the project, where they complete an interview including health and lifestyle questions, a core cognitive assessment, and a self-completed questionnaire of lifetime experiences and physical activity. Of those interviewed, 700 participants aged 18-87 (100 per age decile) continue to Stage 2 where they undergo cognitive testing and provide measures of brain structure and function. Cognition is assessed across multiple domains including attention and executive control, language, memory, emotion, action control and learning. A subset of 280 adults return for in-depth neurocognitive assessment in Stage 3, using functional neuroimaging experiments across our key cognitive domains.

Formal statistical models will be used to examine the changes that occur with healthy ageing, and to evaluate age-related reorganisation in terms of cognitive and neural functions invoked to compensate for overall age-related brain structural decline. Taken together the three stages provide deep phenotyping that will allow us to measure neural activity and flexibility during performance across a number of core cognitive functions. This approach offers hypothesis-driven insights into the relationship between brain and behaviour in healthy ageing that are relevant to the general population.

**Discussion:**

Our study is a unique resource of neuroimaging and cognitive measures relevant to change across the adult lifespan. Because we focus on normal age-related changes, our results may contribute to changing views about the ageing process, lead to targeted interventions, and reveal how normal ageing relates to frail ageing in clinicopathological conditions such as Alzheimer’s disease.

## Background

As greater numbers of us are living longer e.g. [1], it is increasingly important to understand how we can age healthily. Growing older involves changes to most aspects of our lives, but one of the most important changes is to our mental or cognitive health. The aim of the Cambridge Centre for Ageing and Neuroscience (Cam-CAN) project is to identify the neural mechanisms underpinning successful cognitive ageing.

The Cam-CAN protocol has been developed to provide a comprehensive and theoretically-motivated examination of the hypothesis that preserved cognition across the lifespan depends on the brain remaining functionally flexible. This hypothesis is grounded in a growing number of findings demonstrating that older adults’ brains can remain flexible despite structural decline, and that this flexibility can help support successful cognition into old age [2–5]. Despite promising findings, there has been little systematic examination of the role of neural flexibility across the lifespan which could help us to understand the nature of successful cognitive ageing and how best to support it.

We take a multi-disciplinary approach to understanding the relationship of neural flexibility to cognitive success. We employ stratified sampling known from geographical populations, and combine demographic and lifestyle assessment with a wide range of cognitive and neural measures. This approach of sampling widely across individuals and employing deep phenotyping is critical for understanding the potentially complex interactions between brain, cognition, demographic and lifestyle factors that underpin successful cognitive ageing. By providing concrete links between cognitive neuroscience and everyday outcome measures, our approach will help drive targeted interventions.

## Research aims & strategy

### Research questions

Our main aim is to identify the factors that predict successful ageing. We will do this by investigating, in age groups across the adult lifespan, the neural systems underpinning cognitive functions. Our key research questions are:

1. Is successful cognition across the lifespan underpinned by large-scale neural flexibility?
2. What are the relationships between brain and performance across different age groups in adulthood?
3. How do lifestyle and demographic factors relate to cognitive performance and neural integrity at different ages?

### Deep phenotyping

In order to understand the complex brain-behaviour interactions underpinning successful cognitive ageing, we examine brain and behaviour measures across adult age groups, employing a *deep phenotyping* approach with the following key characteristics:

1. We measure performance on a broad range of cognitive domains, including abilities that typically decline with age and those that do not. We do this because (a) different domains may have different relationships to age or be affected differently in different individuals; (b) different domains may interact differentially with age to affect performance in complex multi-componential tasks; (c) combined analyses across domains will help us identify common factors underpinning successful cognition.
2. We employ a multimodal neuroimaging approach, using measures with high spatial resolution (e.g. Magnetic Resonance Imaging; MRI) and high temporal resolution (e.g. Magnetoencepholography; MEG) and measuring multiple aspects of both structure and function. We do this for similar reasons as cross-domain testing, namely (a) different aspects of neural integrity may have different relationships to age; (b) neural structure and function are likely to interact with each other; (c) combined analyses across different measures of function and structure will help identify common factors underpinning neural flexibility. Additionally, (d) multi-domain measures of neural structure and function provide an opportunity for the methodological advancement of ageing research. For example, the use of MEG provides an opportunity to estimate neural activity directly, bypassing any effects of age on the neurovascular coupling that may affect functional MRI (fMRI) measures.
3. We examine demographic, health, and lifestyle factors that give us a unique insight on the link between typical epidemiological measures and multivariate assessments of neural networks, and then examine variation in these relationships across the age groups. This aspect of the Cam-CAN project provides an important way in which the integrity of complex interactive networks can be used to predict everyday outcomes that are important for daily life, and to develop evidence-based interventions to support successful cognitive ageing.

### Integrative analyses

**Figure 1:**
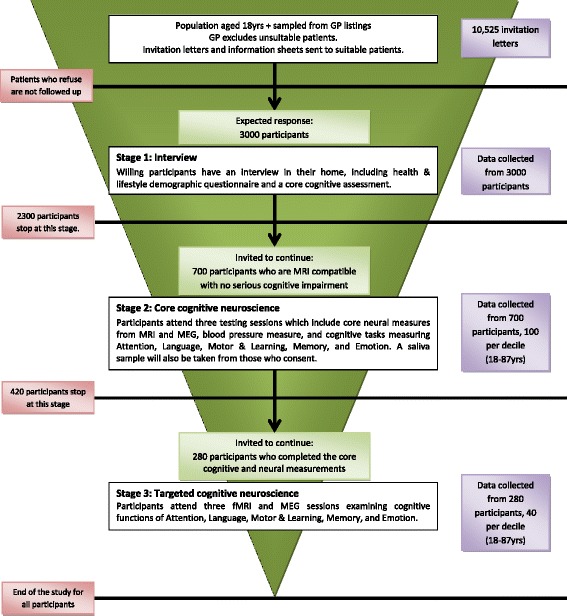
**Flowchart of participant recruitment, with estimates of participant recruitment and overview of participation at each stage.**

Three main stages to the project are described below and in Figure 1. These stages are designed to be integrative rather than progressive or separate. Our analysis approach is likewise interactive, with our major hypotheses based on the relationships between different measures. We will use multivariate neuroimaging methods to test predictions about the neural and cognitive variables that predict successful cognitive ageing. Key to testing hypotheses about neural flexibility are measures of structural and functional connectivity. We predict that preserved cognition across the lifespan depends on maintaining effective neural flexibility in the context of the extensive neural change associated with normal ageing [6,7]. Finally, we will link cognitive and neuroimaging measures to epidemiological measures of demographic background, physical and mental health, and a range of lifestyle and life experience measures. This will enable us to understand the real world implications of neural resilience, and to create targeted interventions that link everyday lifestyle choices to neural flexibility and cognitive health.

## Methods

### Participants: recruitment and selection

See Figure 1 for an overview of study recruitment and participation. In Stage 1, the sampling frame is the primary care population list of residents in particular geographical areas. This is the closest possible sample in the UK to being truly population representative in nature since registration with GP is nearly universal in the UK’s socialised health care system. The sample itself consists of a population-based representative sample of approximately 3000 adults, aged 18 years and over, who are drawn from the general population via Primary Care Trust (PCT)’s lists within the Cambridge City (UK) area. The study is designed to be all inclusive but term-time residents of colleges and universities are excluded, and participants whose Primary Care Physician feel are inappropriate to include will not take part in the study. This study is conducted in compliance with the Helsinki Declaration, and has been approved by the local ethics committee, Cambridgeshire 2 Research Ethics Committee (reference: 10/H0308/50).

Information obtained in the study will be used to link the selected individuals back to the general population as a whole. Background information on the demographics of the populations sampled will be collected from the regional Public Health Observatory which holds aggregated data for the relevant primary care and geographical populations, which will enable us to relate our data to regional and national data. The project researchers are provided with basic contact information from each participating PCT, which is used to send out introductory letters.

From participants in Stage 1, a subset of approximately 700 individuals are recruited into Stage 2, including 100 individuals in each decile age 18-87 (with equal numbers of men and women). Participants must indicate their willingness to continue to the next stage of the project, be cognitively healthy, not have a serious psychiatric condition, meet hearing and English language requirements for experiment participation, and be eligible for MRI scanning (see also the description of exclusion criteria in “Stage 2 screening measures” and Table 1).

**Table 1:** **Cam-CAN exclusion criteria: conditions assessed at stage 1 interview that could result in exclusion, listed by category**

**Exclusion category**	**Exclusion criteria**
**Cognitive health**	MMSE score 24 or less (calculated in interview)
	Missing MMSE scores (assumed to be 24 or less)
	Severe memory defect
	Consent difficulties for next stage
**Communication difficulties**	Hearing problems (difficulty completing interview with hearing aid; inability to hear 35 db at 1000 Hz in either ear in interview functional hearing screening; hearing aid that cannot be removed)
	Insufficient English language (native language not English and not bilingual English from birth)
	Vision difficulties (correct near vision of 20/100 or worse with both eyes)
**Medical problems (self-report of diagnosis)**	Dementia diagnosis/Alzheimer’s Disease
	Parkinson’s disease
	Motor Neurone disease
	Multiple Sclerosis
	Cancer (history of brain tumour or chemotherapy/radiotherapy for any cancer in last 6 months)
	Stroke
	Encephalitis
	Meningitis
	Epilepsy
	Head injury with serious results (coma, unconscious for >2 hrs, or skull fracture)
	Recently diagnosed or uncontrolled high blood pressure
	Pregnancy or trying to become pregnant
	Current serious psychiatric conditions (bipolar disorder, schizophrenia, or psychosis)
**Mobility problems**	Restricted mobility which would prevent further participation
	Inability to walk 10 metres
**Substance abuse**	Past or current treatment for drug abuse
	Current drug usage
	Refusal to answer substance abuse questions
**Specific MRI/MEG safety and comfort exclusions**	Heart operation
	Blood vessel procedure or device (carotid artery vascular clamp; venous umbrella; stent, filter or coil; Swan-Ganz catheter; vascular access ports or catheters)
	Neurostimulator or spinal fusion stimulator
	Electrodes on body, head or brain
	Pump, Implant or pacemaker
	Brain Operation
	Metal splinters in eye, head or ear
	Shrapnel, buckshot or bullet in body
	Wire sutures or surgical staples
	Artificial joints that are MRI incompatible (jaw/maxillary reconstruction; shoulder prosthesis; any other joint replacement surgery in the last 3 months)
	Bone fixation rods or plates in jaw, head, shoulders or spine
	Non-removable dental brace
	Non-removable prosthesis or removable eye prosthesis
	Inability to lie flat for an hour
	Claustrophobia
	Body piercings that cannot be removed
	IUD that is MRI incompatible
	Transdermal delivery patches that cannot be removed
	Tattoos on head face or neck

From participants in Stage 2, a subset of approximately 280 individuals (40 in each sampled decile) are recruited into Stage 3. Participants must be willing to continue, and must still meet all of the eligibility requirements for recruitment into Stage 2.

### Study sampling numbers and power calculations

The size of the study sample population required to reach 100 qualified participants per decile for Cam-CAN Stage 2 is expected to vary by age when accounting for exclusion and refusal, estimated population data, clinical based experience and estimates of individuals who may refuse to participate in neuroimaging. Numbers are adjusted for the proportion of the general population with exclusion criteria including MR safety contraindications (e.g. pacemakers), learning disability (living at home), cognitive impairment (Mini-Mental State Examination (MMSE) [8] score of 24 or less) and reduced response from individuals with limited longstanding illness or disability. Proportions are estimated based on data from the Office of National Statistics (ONS), the Medical Research Council Cognitive Function and Ageing Study (MRC-CFAS) [9] and the National Health Service (NHS) registrations. We assume that only 30% of the population will undertake the initial interview and of those who do, 40-50% will agree to take part in Stage 2 (age dependent). Numbers predicted to be needed for Stage 1 are shown in Table 2. The age group above age 88 are recruited to the same population proportion as the 78-87 decile, in order to enable cohort comparison with other population-based studies and investigation of the rare group of oldest old who are experiencing healthy ageing.

**Table 2:** **Estimated Stage 1 recruitment across the deciles to recruit 100 participants in each decile (age 18-87) for Stage 2**

	**Decile 1 (18-27 years)**	**Decile 2 (28-37 years)**	**Decile 3 (38-47 years)**	**Decile 4 (48-57 years)**	**Decile 5 (58-67 years)**	**Decile 6 (68-77 years)**	**Decile 7 (78-87 years)**	**Decile 8 (88+ years)**
Contact	750	775	850	950	1250	1400	2850	1700
Interview	250	250	275	300	400	450	850	500

Estimates include numbers per decile to be contacted and interviewed.

The Cam-CAN structure provides sufficient sample size in each decile to separate age-related change from other sources of individual variation. A number of different comparisons can hypothetically be undertaken using this structure. All hypotheses are investigated at a power of 80% and α = 0.05: for linear regression, assuming the continuous data are standardised to a N(0,1) distribution, 100 per decile enables us to investigate i) a linear decline of ±0.04 across the age range; ii) a difference in linear regression slope of size ±0.06 between two risk factor groups with a prevalence of 50% (such as gender); iii) differences in the mean values of two groups (defined with 50% prevalence) of ±0.2; iv) for dichotomous outcomes with prevalence of 0.5 in one group to detect a difference of at least ±0.1. This sample is sufficiently large to be able to detect non-linear change with age, such as a change in rate of decline, and the required size to detect stability with age (to exclude a slope of up to ±0.03 per decile). Multiple hypotheses can also be undertaken, such that linear decline of slope 0.1 can be detected for 100 independent investigations protecting the type I error rate (false positives).

## Research method: materials and procedure

### Overview of protocol stages

1. Cam-CAN Stage 1: Interview. *Selection*: Individuals are randomly selected from the GP lists from participating surgeries in Cambridge City. Individuals are checked by the GP for eligibility of participation. All individuals receive a letter informing them that an interviewer will contact them. Those that do not refuse at this stage are visited up to three times at different times of the day by a research interviewer to arrange an appointment. *Consent:* Prior to the interview individuals give written informed consent for the study and record linkage. Individuals who lack the capacity to give consent are not included. Written informed consent is also given by participants at each session for Stages 2 and 3. *Interview:* Research interviewers visit approximately 3000 participants in their homes to complete a single session consisting of a computerised health and lifestyle questionnaire and a core cognitive assessment. Interviewers also collect a self-completed lifetime experiences and physical activity questionnaire that participants receive in advance of the home interview. The interview is designed to act not only as the first core session, but also to provide assessment of eligibility and selection for Stage 2 (see Table 1 for a list of exclusion criteria). The number of individuals needed for the Stage 1 is flexible as the goal is to recruit 100 individuals per sampling decile into Stage 2 (see also Table 2).
2. Cam-CAN Stage 2: Core Cognitive Neuroscience. From participants completing Stage 1, we select 700 individuals who pass the eligibility criteria to participate in Stage 2 (see Table 1), including 100 individuals from each decile (18-87 years old). Participants complete a series of cognitive experiments in three testing sessions (see Table 3 for overview of session contents). During one session we also collect core structural and functional MRI measures, and during another session we collect core MEG measures. Participants also supply a saliva sample for genotyping, and have basic physiological measures taken (height, weight, blood pressure).
3. Cam-CAN Stage 3: Targeted Cognitive Neuroscience. From participants completing Stage 2, we then select 280 individuals to participate in Stage 3, including 40 individuals from each decile (18-87 years old). Participants return for targeted neuroimaging experiments across a range of cognitive domains in two MRI and one MEG session (see Table 4 for overview of session content and Table 5 for an overview of how participants are allocated to sessions). At this stage participants repeat cognitive screening and physiological measures.

**Table 3:** **Stage 2 session content and timing, listed by category**

**Session**	**Measure**	**Modality**	**Approx. duration (mins)**
**Session 1**			
	Weight	Physiological	2
	Height	Physiological	2
	Blood Pressure	Physiological	11
	Visual short-term memory	Behavioural	35
	Emotion expression recognition	Behavioural	20
	T1-weighted structural image	MRI	5
	T2-weighted structural image	MRI	5
	Diffusion-Weighted Images	MRI	10
	Magnetisation Transfer Ratio images	MRI	5
	Resting state T2*-weighted	fMRI	9
	Movie watching T2*-weighted	fMRI	8
	Sensorimotor task T2*-weighted	fMRI	9
	Field maps	fMRI	1
**Session 2**			
	Face recognition: familiar faces	Behavioural	10
	Face recognition: unfamiliar faces	Behavioural	10
	Fluid Intelligence	Behavioural	20
	Hotel task	Behavioural	20
	Sentence comprehension	Behavioural	30
	Resting state	MEG	9
	Sensorimotor task	MEG	12
**Session 3a**			
	Emotional reactivity and regulation	Behavioural	55
	Force matching	Behavioural	14
	Motor learning	Behavioural	21
	Picture-picture priming	Behavioural	20
	Proverb comprehension	Behavioural	5
	Tip-of-the-tongue	Behavioural	10
**Session 3b**			
	Emotional memory	Behavioural	90
	Picture-picture priming	Behavioural	20
	Proverb comprehension	Behavioural	5
	Tip-of-the-tongue	Behavioural	10

**Table 4:** **Stage 3 session content and timing, listed by category**

**Session**	**Measure**	**Modality**	**Approx. duration (mins)**
**fMRI session 1**	Weight	Physiological	2
	Height	Physiological	2
	Blood Pressure	Physiological	11
	T1-weighted structural image	MRI	5
	T2- weighted FLAIR structural image	MRI	5
	Arterial Spin Labelling	fMRI	4
	Emotional expression recognition	fMRI	15
	Free selection	fMRI	12
	Fluid Intelligence	fMRI	7
	Stop-Signal, Go/No-Go	fMRI	22
**fMRI session 2**	Weight	Physiological	2
	Height	Physiological	2
	Blood Pressure	Physiological	11
	T1-weighted structural image	MRI	5
	T2-weighted FLAIR structural image	MRI	5
	Arterial Spin Labelling	fMRI	4
	Fluid Intelligence	fMRI	7
	Picture naming	fMRI	15
	Sentence comprehension	fMRI	32
**fMRI session 3**	Resting state	fMRI	5
	Emotional Memory encoding	fMRI	25
	Emotional Memory test	Behavioural	60
**fMRI session 4**	Resting state	fMRI	5
	Emotional reactivity and regulation	fMRI	30
	Visual short-term memory	fMRI	35
	Field maps	fMRI	1
**MEG session 1**	Mini Mental State Exam	Behavioural	7
	Hearing assessment	Sensory	4
	Vision assessment	Sensory	2
	Resting state	MEG	5
	Incidental memory	MEG	10
	Multi-mismatch	MEG	18
	Stop-Signal, Go/No-Go	MEG	30
**MEG session 2**	Mini Mental State Exam	Behavioural	7
	Hearing assessment	Sensory	4
	Vision assessment	Sensory	2
	Resting state	MEG	5
	Picture naming	MEG	20
	Sentence comprehension	MEG	18
	Word recognition	MEG	10

**Table 5:** **Stage 3 Session combinations across participant groups**

	**fMRI sessions**		**MEG session**
*Group 1 (N = 70)*	fMRI Session 2	fMRI Session 3	MEG Session 2
*Group 2 (N = 70)*	fMRI Session 1	fMRI Session 3	MEG Session 1
*Group 3 (N = 70)*	fMRI Session 1	fMRI Session 4	MEG Session 1
*Group 4 (N = 70)*	fMRI Session 2	fMRI Session 4	MEG Session 2

Contents of fMRI Sessions and MEG Sessions are listed in Table 4. Each participant returns for two fMRI sessions and one MEG session.

### Cam-CAN Stage 1: interview

The Stage 1 interview takes place in participants’ homes and in the main takes the form of an interview, with interviewers asking questions and recording responses using a computerized script. Exceptions to this that required additional equipment or testing materials include assessing vision, hearing, and basic reaction time. Equipment and testing materials for individual measures are described below. Participants also provide additional information in the form of a written questionnaire that is posted to them before the interview, and is described below.

#### Stage 1 materials

1. Demographic information
  Interviewers record basic participant information including date of birth and gender, and measure participant characteristics that may affect experimental measures including handedness, as assessed by the Edinburgh Handedness Inventory [10]. Detailed demographic information is gathered from participants including information on marital status, accommodation, employment, income, education, birthplace, ethnicity, and English language history. Additional detailed information about education and training, travel, hobbies, and social activities is gathered using a self-completed questionnaire using items from The Lifetime of Experiences Questionnaire (LEQ) [11] and the European Prospective Investigation into Cancer Study-Norfolk Physical Activity Questionnaire (EPIC-EPAQ2) [12]. Different versions of the questionnaire are used for young (18-29), middle-aged (30-64), and older (over 65) participants. The versions for middle-aged and older participants also ask for employment, education, and other information from earlier periods in their lives.
2. Cognitive functioning
  The cognitive assessment for all participants includes the MMSE [8], the Addenbrooke’s Cognitive Examination (ACE-R) [13], logical memory from the Weschler Memory Scale Third UK edition (WMS-III UK) [14], Spot the Word [15], and The Cambridge Memory Questionnaire (the Cambridge 10MQ), a set of 10 questions probing whether participants have memory problems. Spot the Word was time-limited to 5 minutes due to interview time restrictions but this was not indicated to the participants and interviewers could be flexible. Taken together, these measures assess overall cognitive health as well as a number of targeted processes including memory, verbal fluency, premorbid IQ, basic language function, and visuospatial abilities.
3. Response time measurements
  A “simple” response time task (SRT) and a “choice” response time task (CRT) assess basic aspects of speeded responses. In the SRT, participants view an image of a hand with blank circles above each finger, while resting their right hand on a response box with four buttons, one for each finger. When the index finger circle turns black on the image, they press with their index finger as quickly as possible. On pressing the button (or after maximum 3 seconds), the circle becomes blank again, and the variable inter-trial interval (ITI) begins. The ITI varies pseudo-randomly with positively skewed distribution, minimum 1.8 seconds, mean 3.7 seconds, median 3.9 seconds, and maximum 6.8 seconds. There are 50 trials, and the principle outcome measure is the reaction time from stimulus onset to button press. In the CRT, timing parameters are the same as for SRT, but on each trial any one of the four circles above the fingers could become black, and the participant must press the corresponding finger as quickly as possible (maximum 3 seconds response time). There are 67 trials, and the principle outcome measures are the reaction time from stimulus onset to button press (averaged across all four fingers) and the rate of commission errors (pressing an incorrect button).
4. Social contact
  In addition to questions elsewhere about social relationships (e.g. marital status), and activities (e.g. hobbies), interviewers ask a number of targeted questions about social support including communications with friends and relatives, participation in community, religious, or social organisations, and whether or not participants have children.
5. Measures relevant to physical health, disease, and frailty
  A number of measures are taken to assess the physical health of participants, as well as behaviours that are likely to affect their health. Participants provide self-rated assessment of their general health, family history of specific health problems (e.g. heart disease, stroke, and diabetes), and a self-report of their own experience of specific health conditions including those relevant to cardiovascular health (high blood pressure, high cholesterol, etc), mental health (e.g. depression, insomnia), cancer, and a range of other serious health conditions.
  Participants are also asked about their history of falls, and perform two tests of balance: first they balance on one leg for 30 seconds, with their eyes open and then with their eyes closed; second, they perform five chair rises from a seated position.
  In addition to self-reports of hearing and vision problems, participants receive screening measures of hearing and vision. The Siemens HearCheck Screener tests participants’ hearing for three sound pressure levels (75 dB SPL, 55 dB SPL, and 35 dB SPL) at two frequencies (1000 Hz and 3000 Hz). The test is performed without hearing correction (i.e. hearing aids) to mimic conditions in the MRI and MEG sessions of Stage 2 and Stage 3. In order to test near vision, a version of the Snellen test [16] is performed with corrected vision (i.e. glasses) if necessary.
  To assess specifics of sleep disturbances, participants complete the Pittsburgh Sleep Quality Index (PSQI) [17]. This measure was designed for purposes of assisting diagnosis of sleep disorders, and provides seven subcomponents of sleep quality as well as an overall score of sleep disturbance.
  Finally, participants provide details of medication that they are currently taking, including all prescription and over-the-counter medication.
  In addition to objective and self-report measures of physical health, participants answer questions about a number of health-related behaviours. These include questions about current and past alcohol and tobacco use, as well as drug use as assessed by the Drug Abuse Screening Test (DAST-20) [18]. Participants provide information on aspects of their diets (e.g. servings of vegetables per day, frequency of eating processed meats), and a detailed description of their habitual physical activity using the EPIC-EPAQ2 [12].
6. Mental health
  Mental health is assessed by participants’ self-report of whether they had been diagnosed and treated for anxiety or depression and at what age. Participants also complete the Hospital Anxiety and Depression scale (HADS) [19], which provides an aid to diagnosis and continuous measures of the severity of symptoms of anxiety and depression.
7. Measures for older adults
  Most measures are taken from all participants across the age range. Two additional metrics are taken to allow for direct comparison with the older cohort of participants in MRC-CFAS [9]. First, participants older than 65 years are asked a series of nine questions probing their historical memory knowledge, with questions taken from the Cambridge Cognitive Examination (CAMCOG) [20]. Second, participants older than 65 years complete a series of 33 questions about activities of daily living.
8. Questionnaire consistency with other UK Population-Based Studies
  The questionnaire is designed to overlap with other population-based studies. Demographic information, diet and working experiences are the same as used in the UK Census for 2011 and UK Biobank, and the cognitive battery for the older population and the activities of daily living is the same as in the MRC-CFAS I and II, augmented with a larger number of activities from the Cambridge City over 75 Study (CC75C) and EPIC-Norfolk. Additional information on substance usage is taken from the ROOTS project [21] as well as other published scales as detailed above.

#### Stage 2 screening measures

A number of measures from the Stage 1 home interview are used to screen participants for further participation. A full list of exclusion criteria are detailed in Table 1, and to continue to Stages 2 and 3, participants must be willing to participate and not have any exclusion criteria. In brief, participants who continue must meet the following criteria:

1. Be cognitively healthy, with MMSE scores above 24.
2. Not have MRI safety, comfort, or medical contraindications, such as having various kinds of non-MRI compatible medical implants (cardiac pacemaker, cochlear implants, etc.) or magnetic foreign objects (e.g. shrapnel) close to the head, being pregnant, being claustrophobic, or being unable to lie still for the necessary length of time (approximately one hour). Criteria for MRI and MEG participation are defined by Standard Operating Procedures set by the Medical Research Council Cognition and Brain Sciences Unit (MRC-CBSU).
3. Not have MEG contraindications that could affect data collection, such as extensive dental work (e.g. permanent brace).
4. Not have other conditions including serious head injury, current drug abuse, or current serious psychiatric condition (e.g. bipolar, schizophrenic).
5. Not have poor hearing which could affect the ability to participate in experiments (failing to hear 35 dB in either ear).
6. Not have poor English or English which is extremely subordinate to another language, which could affect participation in experiments (i.e. those whose native language is not English or who are not bilingual English-speakers from birth).

### Cam-CAN Stage 2: core cognitive neuroscience

#### Design

Stage 2 measures include 14 behavioural tasks, core MRI measures, core MEG measures, physiological measures including height, weight, and blood pressure, and a saliva sample. Materials and procedures for each task are described below. Measures are divided into four testing sessions, and each participant attends three out of four of the sessions. The content and timing for each session is described in Table 3. All participants attend Sessions 1 and 2, and either Session 3a or 3b, with the order of the sessions not controlled.

#### Cognitive/behavioural tasks

Fourteen behavioural tasks are used to assess cognitive processing across five core cognitive domains: executive function, emotional processing, motor and action function, language processing, and memory. Some tasks have been adapted from standardized tasks and measures with clinical significance, and many were experiments designed to assess targeted cognitive processes and normal individual variation. Most tasks are primarily from a single domain, but many involve complex processes that combine multiple domains. Tasks are primarily a mixture of paper-and-pencil tasks and simple computerized experiments conducted on a laptop computer, although some tasks required specialist equipment (e.g. Motor learning and Force matching) or were table top tasks (e.g. the Hotel task).

1. Emotion expression recognition
  This task was developed to tap into processes underpinning the recognition of emotional expressions [22]. This ability represents a potentially dynamic interaction between age-related declines in recognizing facial expressions of emotion [23,24] and age-related stability or even improvement in many aspects of emotional processing [25,26]. Likely neural regions contributing to the decline in facial expression recognition include the ventral prefrontal regions, which are connected to other regions involved in facial expression processing, such as the amygdala.
  The experiment involves morphed facial continua ranging between the following six facial expression pairs included in the Ekman and Friesen [27] pictures of facial affect series: happiness-surprise, surprise-fear, fear-sadness, sadness-disgust, disgust-anger, and anger-happiness (see [22] for description of materials construction). The stimulus set consists of 15 practice images and 30 experimental images which are repeated in random order across five experimental blocks. For each trial an individual morphed picture is presented on a computer monitor for three seconds. Participants then have as much time as needed to choose which of the six emotion labels (happy, sad, anger, fear, disgust, or surprise) best describes each facial expression. Performance is based only on trials where the morphed image is biased 70% or 90% towards one expression, which provides total accuracy maxima of 20 for each of the six expressions (happiness, surprise, fear, sadness, disgust, and anger).
2. Emotional memory
  This task provides a multidimensional assessment of several aspects of both explicit and implicit memory, and how they are affected by emotional valence. Memory problems are amongst the best-documented declines in normal ageing, but not all aspects of memory are equally affected, with explicit recollection declining most precipitously, recognition-based familiarity judgments being less seriously affected, and aspects of repetition priming often being preserved across the lifespan [28–30]. These different aspects of memory are also underpinned by different neural systems, with medial temporal lobe (MTL) critical for explicit memory, and sensory systems such as occipitotemporal cortex involved in (visual) priming.
  Furthermore, it is well-established that memory is generally superior for emotional relative to neutral stimuli, which is often attributed to modulation of MTL by the amygdala. This emotional memory advantage may be affected by age, in particular if older adults are increasingly biased towards positively-valenced material, as has been suggested [26]. The task employs a 3 × 3 factorial design, with three types of memory (priming, familiarity, recollection) crossed with three types of valence (positive, neutral and negative).
  Like many studies of memory encoding, this task employs a Study phase followed by a Test phase. The Study phase includes 120 trials, and for each trial participants see a background picture for 2 seconds, after which a foreground picture of an object is superimposed. Participants are instructed to imagine a “story” linking the background and foreground picture, and after an 8 second presentation, the next trial begins. The emotional valence manipulation affects only the background image, which is negative, neutral, or positive. Background images are selected from the International Affective Pictures set (IAPS) [31] while all foreground objects are neutral. The Test phase is unannounced (i.e. encoding is incidental), and begins approximately 10 minutes after the end of the Study phase. This phase consists of 160 trials, where 120 trials involve the studied foreground objects, and 40 trials involve new, unstudied objects. Each trial assesses three aspects of memory in succession: visual priming, recognition, and recollection of the background image, including identity and valence. Participants first identify a visually-degraded object presented for 1000 milliseconds (to index perceptual priming). They then see an intact version of the same object which remains on the screen while they indicate their confidence about whether or not they had seen the object in the Study phase (to index familiarity, or “item memory”). Finally, while the object is still displayed, they are required to say whether the valence of the background image against which that object was seen in the Study phase was positive, neutral or negative, and then to provide a verbal description of that background image (to index recollection, or “associative memory”).
  Priming is assessed by comparing accuracy of naming degraded objects that were studied versus not studied. Recognition confidence is transformed to an accuracy measure using signal-detection theory. Background valence recall is scored as accurate or not by the experimenter, and recollection of the background identity is scored on a 4-point scale by the experimenter (-1 = incorrect, 0 = no information given, 1 = gist correct, 2 = detail correct). Importantly, priming, recognition (when collapsing confidence) and valence recall can also all be scored by the same metric of “Pr”, the probability of hits minus probability of false alarms (where 0 is chance and 1 is perfect discrimination), which renders the three memory scores commensurate.
3. Emotional reactivity and regulation
  This task measures processes involved in regulating emotional responses, a critical everyday function which relies on extensive cortical and subcortical networks, including prefrontal cortex, amygdala, insula, and ventral striatum [32]. Functional imaging studies implicate prefrontal cortex in the top-down regulation and modulation of subcortical regions (e.g. amygdala) involved in the core emotion response [33]. While there is evidence for age-related reduction in prefrontal cortex [6], there is behavioural evidence that emotion regulation is preserved or even improves in older adults [25,26,34]. Thus, emotion regulation is an important domain for examining age-related flexibility, especially because emotions interact reciprocally with other mental processes, such as attention and memory.
  In this task, participants view positive, neutral, and negative film clips and rate their emotional responses after each. For some of the negative films they are asked to reappraise the film content by reinterpreting its meaning in order to reduce the emotional impact. The experiment consists of eight experimental blocks, each containing four experimental trials from one of the four main conditions: (1) watch neutral, (2) watch positive, (3) watch negative, or (4) reappraise negative. For each trial, participants received a prompt to indicate the valence and how they should observe the film (e.g. “WATCH NEUTRAL” or “REAPPRAISE NEGATIVE”). This is followed by a 30 second film clip. Following the film clip, participants provide ratings on three scales. First, they rate how negative they felt during the film from 0 to 100. Second, they rate how positive they felt during the film from 0 to 100. Finally, they use a continuous scale to provide a “strategy” rating, indicating the degree to which they had been watching versus reappraising the film. Each scale appeared for 10 seconds. For blocks containing positively or negatively valenced films, after the four experimental trials there is a “washout” clip where participants watch a calming 30-second film clip before the next block begins.
  This task provides measures of how the positive and negative films affect the viewer during the “watch” condition. These “emotional reactivity” scores are calculated by comparing the ratings during the positive and negative films to those from the neutral films. Additionally, this task provides a measure of how well people regulate their negative emotions in the negative reappraise condition. This “emotional regulation” score is calculated by comparing the watch and reappraise conditions for the negative films.
4. Face recognition: familiar faces
  The Face recognition task employs familiar faces (of public figures) to assess the ability of participants to recognize people from their pictures. Previous research has shown evidence of an age-related decline in face recognition [35,36], which may be associated with age-related memory declines. While the Face recognition test of unfamiliar faces examines novel face processing, this task measures the ability to recognize known (famous) faces.
  Recognition of familiar faces is assessed with pictures of 30 celebrities’ faces intermixed with 10 unfamiliar face foils. The faces are presented individually in a pseudo-random order. For each face, participants are asked first whether the person is familiar; if so they are asked to provide identifying information such as their occupation, nationality, or work for which they are known. Third, participants provide the name of each familiar person. After the set of 30 faces is complete, the participants are re-presented with any public figures that they did not recognise as “familiar” or any “familiar” people for whom they could not provide identifying information. Participants are provided with the picture and name, and again asked to try to provide their occupation and additional identifying information, in order to provide an additional measure of familiarity.
  Separate scores are calculated for the proportion of famous faces where the participant (1) recognises the face as familiar, (2) provides unique identifying information, and (3) provides their correct full name. In addition, a score out of 10 is given to the number of unfamiliar faces that each participant correctly identifies as unfamiliar. Proportional scores are calculated using each participant’s individual maximum: the total maximum of 30 familiar faces is adjusted to remove any faces that were persistently unfamiliar in the second phase where the picture is re-presented with the name.
5. Face recognition: unfamiliar faces
  The Benton Test of Facial Recognition [37] assesses the ability to match pictures of unfamiliar faces. Previous research has shown evidence of an age-related decline in face recognition [35,36]. While the Face recognition test of familiar faces assesses recognition of public figures, this task assesses the ability to recognize a newly-seen face.
  We use the short form of the Benton Test of Facial Recognition [38]. There are 27 trials, and on each trial the participant is shown a target face and array of six faces. The task is to find one or more examples of the target face amongst the array of six. For the first six trials the participant has to find one example of the target face in the array of six, in the following seven trials he/she is required to find three examples of the target. Changes in head orientation and lighting can occur between the target and array faces. Each correct response is assigned a score of 1, so the total score is recorded out of a possible score of 27.
6. Fluid intelligence
  Fluid intelligence is an important central cognitive measure because of its broad positive correlations with other cognitive tests. The hypothesis is that, in large part, fluid intelligence reflects the function of the frontoparietal multiple-demand system (see [39]) in constructing the mental control program for any form of complex activity.
  We use the standard form of the Cattell Culture Fair, Scale 2 Form A [40,41]. The test contains four subtests with different types of nonverbal “puzzles”: series completion, classification, matrices, and conditions. Each subtest is timed although participants are not informed about precise timings beforehand, with 3 minutes for the first subtest, 4 minutes for the second, 3 minutes for the third, and 2.5 minutes for the final subtest. Before each subtest, instructions are read from the manual and participants are given examples.
  The Cattell test is a pen-and-paper test where the participant chooses a response on each trial from multiple choices, and records responses on an answer sheet. Correct responses are given a score of 1 for a total maximum score of 46.
7. Force matching
  The Force Matching task examines the integration of predictive signals in motor control. Normal motor control relies on an integration of predictive signals (the predicted result of one’s own action) with low-level sensory signals (sensory feedback from the moving body part). This integration leads to an ‘attenuation’ of the perceived sensory intensity, such that the result of an action that is self-caused is perceived as less intense than a similar sensory event that is externally caused.
  The Force Matching task measures the extent of attenuation in order to look into whether and how sensorimotor integration changes during normal ageing. A significant contribution to the morbidity and mortality of the healthy ageing population comes from the gradual deterioration of motor behaviour, and this may reflect both peripheral changes in muscles and joints, and deterioration in the function of the central nervous system.
  During this task participants experience a target force on their left index finger and then use their right index finger to match (reproduce) the target force. On each trial, a lever attached to a torque motor applies a target force to the left index finger, which rests under the lever. The target force is applied for 2.5 seconds, with 2.5 seconds of ramping up and ramping down before and after the application. An auditory beep sounds and a visual prompt appears on the computer screen, after which participants match the target force for a period of 4 seconds. There are two ways that participants attempt to match the target force, creating the two main experimental conditions for this task. In the Direct condition, participants press directly on top of the lever with their right index finger, mechanically transmitting the force to the left finger resting below the lever. In the Slider condition, participants move a slider to indirectly transmit force to the left finger via the torque motor and lever. A force sensor at the end of the lever measures both the target and matched forces applied to the left finger. All participants perform both the Direct and Slider conditions and the order is counterbalanced across participants.
  For each condition, an initial familiarization of eight trials (two cycles of the four target forces) is performed. The main experiment for each condition consists of 32 trials (eight cycles). Data from each condition of the main experiment is analysed by calculating the average difference between the target force and the matched force on each trial (measured by the force sensor) across the four target force levels. This is referred to as overcompensation, and is positive if participants produced larger matching forces relative to the target forces.
8. Hotel task
  This task examines aspects of executive function that are important for complex planning and multitasking [42]. These abilities are linked to anterior frontal cortex, and have been dissociated from another aspect of executive function, namely fluid intelligence (see Fluid intelligence task description above).
  The Hotel task is so named because participants are asked to imagine that they are a manager of a hotel with several tasks to perform. This is a table-top task, and employs props to allow participants to perform a set of five fictionalized tasks: writing out customer bills, sorting money from a charity collection, proofreading an advertising leaflet, sorting mixed playing cards, and alphabetizing name labels for a group of conference attendees. Materials for the five tasks are laid out on a table.
  Participants are asked to spend 10 minutes engaged in the tasks, dividing their time between all five tasks. Critically, there is insufficient time to complete any task so that the participant must spontaneously organize their time to ensure they sample all tasks. There is a clock available so participants can check the time when they wish, and if a participant spends more than 5 minutes on the first task they are given a single verbal reminder that the aim of the experiment is to try all five of the tasks. Experimenters record the time spent on each task.
  Optimal multitasking would mean that during the 10 minute experiment, all five tasks were attempted, for 2 minutes each. Key outcome measures include how many of the five tasks were attempted and the total deviation from optimal time allocation of 2 minutes per task.
9. Motor learning
  This task taps into motor adaptation, the process of learning new kinematic control in response to deviations in a voluntary action. This online control requires integrating predictive signals for an expected outcome with the actual movement outcome. Learning occurs in response to an unexpected outcome of an action, by updating the model the brain has about the dynamic properties of the environment. This model updating occurs throughout life and is a hallmark of neuroplasticity.
  In this task participants use a stylus to make movements to targets. Targets are displayed on a monitor which is projected horizontally and stylus movements are recorded using a digitising touch pad. On each trial participants view the horizontal display and see a yellow target disc 5cm from a central point and in one of four positions. The participants’ task is to move the stylus to hit the target within 800 milliseconds, or they receive an error tone and “Too slow” displays. After practicing with their hands visible, during the main experiment a cardboard occluder prevents participants from seeing their hand, but they can see the position of the stylus represented on the display as a red cursor.
  The main experiment consists of a total of 192 trials divided into three phases. During the pre-exposure phase, participants perform 24 trials in which the red cursor accurately represents the position of the stylus (veridical condition). During exposure phase, participants perform 120 trials in which the position of the cursor is rotated 30° clockwise relative to the central position. Participants must adapt in this perturbed condition in order to hit the target. During the final post-exposure phase, participants perform 48 trials in the veridical condition; to the extent that participants adapt during the expose phase, they must now adapt back in the post-exposure phase in order to hit the target.
  There are two main performance measures, movement time to hit the target, and movement trajectory error. The trajectory error is calculated as the difference between the target angle and the angle of the initial movement trajectory. Both measures are averaged across five distinct periods of the experiment: the pre-exposure phase, late and early in the exposure phase, and late and early in the post-exposure phase. Examining how movement time and movement trajectory error change across the five periods provides measures of initial performance, adaptation rate, and re-adaptation rate.
10. Picture-picture priming
  The aim of this task is to assess core processes involved in word production by measuring the effect of phonological and semantic priming on object naming speed and accuracy. Normal ageing is associated with declines in word finding, which have been variously interpreted as due to age-related declines in semantic memory or phonological access. Aspects of word production are also associated with core aspects of attention, so that we expect naming ability to reflect the interaction between both language-specific and domain-general neural systems.
  The experiment involves two phases, a “baseline” phase and a “priming” phase. In the baseline phase participants name aloud a series of 200 pictures of common objects with short (one or two syllable) names, presented in a pseudorandom order. On each baseline trial, a fixation point is presented for 500 milliseconds, followed by an object for 750 milliseconds, followed by a blank screen for 1000 milliseconds.
  In the priming phase, 100 of the baseline pictures are repeated, each preceded by a unique prime object that is either unrelated, phonologically-related, or semantically-related to its target. Phonologically-related pairs overlap in their initial phonemes, having either *high overlap* (first two phonemes, e.g. pencil-penguin) or a *low overlap* (first phoneme, e.g. robin-ruler) between the prime and target phonology. Likewise, semantically-related primes are category coordinates of the target that had been previously rated as having either higher semantic relatedness (e.g. rabbit-squirrel), or lower relatedness (e.g. box-shelf). Finally, unrelated pairs are neither phonologically- nor semantically-related (e.g. frog-kite). On each priming trial, a 500 milliseconds fixation is replaced by the prime picture for 750 milliseconds and 1000 milliseconds of blank screen, followed by the target picture for 750 milliseconds and a blank screen for 2500 milliseconds.
  For both baseline and priming phases, participants are instructed to name every picture as quickly and accurately as possible. Their responses are recorded to a digital sound file as well as scored for accuracy by an experimenter. The primary measures of interest from the baseline phase are correct naming speed and naming accuracy scored out of 200 total possible. Priming effects are calculated by comparing baseline and priming naming times across the unrelated, phonologically-related, and semantically-related conditions.
11. Proverb comprehension
  This task assesses aspects of executive function including abstraction, which are linked to anterior frontal cortex function, and have been dissociated from another aspect of executive function, namely fluid intelligence (see Fluid intelligence task description above).
  This simple task has been included in previous batteries of bedside patient assessments [43]. We used a modified version with materials presented on a computer screen and recorded digitally to improve large-scale data collection. In this task participants provide the meaning of three common proverbs in English: “One swallow does not make a summer”, “Still waters run deep”, and “A bird in the hand is worth two in the bush.” On each trial, a proverb appears written on the screen and participants provide a meaning, in their own words, and then press a key to advance to the next trial.
  Participants’ responses are scored by experimenters as incorrect or a “don’t know” response (0), partly correct but literal rather than proverbial (1), or fully correct and abstract (2). Total scores for each participant are therefore out of six.
12. Sentence comprehension
  The aim of this experiment is to investigate the on-line comprehension of spoken sentences, focussing on syntactic and semantic processing. Previous research suggests age-related preservation of core aspects of language comprehension, which are largely underpinned by a left-lateralised fronto-temporal network.
  The sentence comprehension task is a modified version of similar tasks that have been used with healthy participants and patients with acquired brain damage [44,45]. This task examines core aspects of sentence comprehension by using syntactic and semantic ambiguity, which occurs frequently and naturally in spoken language. Syntactically-ambiguous phrases such as “landing planes” have at least two different syntactic structures, and semantically ambiguous phrases such as “injured calves” have at least two different meanings.
  Although many words and phrases in English are ambiguous in isolation, during comprehension ambiguities are typically disambiguated by the surrounding context. In this experiment the ambiguous phrases are *biased*, so that there is one interpretation that is most frequent. For example, the *dominant* meaning of “injured calves” refers to young cows, and the *subordinate* interpretation refers to leg muscle. Comparing processing during dominant versus subordinate resolutions reveals key processes involved in the activation, integration, and re-evaluation of semantic and syntactic representations. For example, in previous research, healthy participants are slower to accept and more likely to reject subordinate compared to dominant interpretations of syntactic ambiguity, while patients with syntactic impairment are insensitive to the difference between subordinate and dominant interpretation [45].
  Experimental materials include 126 grammatically correct sentences, and 98 grammatically incorrect sentences, which act as control stimuli. Of the grammatically correct sentences, 42 are unambiguous, 42 contain syntactically-ambiguous phrases and 42 contain semantically-ambiguous phrases. For the sentences with ambiguities, each ambiguous phrase is followed by a disambiguating word (e.g., “injured calves *moo*…” or “landing planes *are*…”). Half of the disambiguating words are consistent with dominant interpretation and half with the subordinate interpretations.
  Participants listen to each sentence read in a female voice up to and including the key ambiguous or unambiguous phrase, e.g. “Tom noticed that *landing planes…”.* Two hundred milliseconds after this, participants hear the disambiguating continuation word read in a male voice (e.g. “are”) and have 4 seconds to respond with a button press indicating whether they judge the continuation as acceptable or unacceptable.
  The total set of 224 sentences is presented in one pseudo-random order in two blocks. Participants have six practice trials before the main experiment, and each block also begins with two lead-in trials that are not analysed. Key dependent measures include the proportion of “unacceptable” judgments and the response times in each condition.
13. Tip-of-the-tongue task
  The aim of this experiment is to examine the common word finding failure known as tip-of-the-tongue states (TOTs). Older adults report word finding problems as one of their main concerns in getting older [46,47], because of the perceived link between “forgetfulness” and general cognitive decline. However, previous research suggests that TOTs are a linguistic problem, reflecting temporary failures to map active semantic representations onto phonological representations during lexical production e.g. [48]. Neuroimaging data also suggests a role of domain-general systems important for error monitoring [49], suggesting that successfully avoiding or resolving word finding problems relies on an interaction between language-specific and domain-general processing.
  The current experiment employs pictures of public figures to elicit TOTs. The task includes 50 faces of people from various walks of life (e.g. actors, musicians, politicians, etc), presented in a single pseudorandom order. Participants are instructed to name each person if they can. If they do not know the name of the person (even if the face is familiar), they respond “Don’t Know”. If they are sure they know the name but cannot retrieve it, they respond “TOT” to indicate they are in a tip-of-the-tongue state. For each trial, participants view a 1000 milliseconds fixation which is replaced by a picture which remains on the screen for 5000 milliseconds. Participants either name the person (Know response), say they do not know the name of the person (Don’t Know response), or say they are having a tip-of-the-tongue (TOT response).
  Key measures include the proportion of correct Know responses, Don’t Know responses, and TOT responses. Other response categories include incorrect Know responses, null responses (where the participant gives no response), and trials where participants provide information other than the name (such as semantic information).
14. Visual short-term memory
  This task assesses the processes underpinning visual short-term memory (VSTM). Only a handful of objects can be simultaneously attended to or held in VSTM, and this capacity limit declines with normal ageing [50]. Moreover, VSTM capacity is predictive of domain-general cognitive abilities e.g., [51]. Neurally, the capacity limit is reflected in posterior parietal cortex [52,53], although VSTM tasks activate a frontal-parietal “multiple demand” network [54], and visual memories can be decoded from occipital cortex [55,56].
  This task is a modified version of a previous experiment that separates measures of VSTM quantity and quality [57]. Short term memory for colours is tested using a continuous colour report paradigm [57]. Participants try to remember the colour of circular discs that are presented briefly on a computer screen. After a brief delay, they report the colour of a cued disc, by selecting from a colour wheel that displays a rainbow of hues.
  On each trial, participants see a display for 250 milliseconds which contains a central fixation and one to four coloured discs, with the colours chosen at random. The locations of the discs on the screen are randomly selected from eight points equidistant from a central fixation. Following the brief encoding display there is a 900 millisecond blank screen, and then one of the disc locations is highlighted with a border and the response colour wheel appears. On half of trials, any uncued discs also reappear, to provide the context within which the disc was encoded.
  Participants try to remember the colour of the disc in the highlighted location, and use a touch screen to press their choice on the colour wheel. They indicate their confidence in the selected colour by the length of time they hold down their finger: as they hold their finger down for longer, white confidence intervals spread out around the selected point indicating more uncertainty about their selection. The response interval does not have a time limit: after participants have confirmed their response there is an 830 milliseconds fixation period before the next trial begins.
  After a brief practice, participants complete two blocks of 112 trials, with set-size and probe context being counterbalanced and randomly intermixed within each. Participants also complete a perceptual control block of 56 trials, where single discs are presented at fixation along with the colour wheel, until the participant matches their hue by selecting the appropriate point on the surrounding wheel.
  Measures of VSTM quantity and quality can be estimated by fitting the error distribution with a mixture model approach developed by Zhang and Luck [57]. Estimated parameters include VSTM capacity (K), the accuracy of the reported hues (precision), and the probability of mistakenly reporting an un-cued item [58].

#### MRI session

MR measures are gathered in a one-hour session conducted at the MRC-CBSU on a 3T Siemens TIM Trio System, employing a 32 channel head coil.

Measures in the MRI session include:

1. T1-weighted structural image
  A high resolution 3D T1-weighted structural image is acquired using a Magnetization Prepared RApid Gradient Echo (MPRAGE) sequence with the following parameters: Repetition Time (TR) =2250 milleseconds; Echo Time (TE) =2.99 milliseconds; Inversion Time (TI) =900 milliseconds; flip angle =9 degrees; field of view (FOV) =256mm x 240mm x 192mm; voxel size =1mm isotropic; GRAPPA acceleration factor =2; acquisition time of 4 minutes and 32 seconds.
2. T2-weighted structural image
  A high-resolution 3D T2-weighted structural image is acquired with a SPACE sequence [59] with the following parameters: TR =2800 milliseconds; TE =408 milliseconds; FOV =256mm × 256mm × 192mm; resolution =1mm isotropic; GRAPPA acceleration factor =2; acquisition time of 4 minutes and 30 seconds.
3. Diffusion-Weighted Images (DWI)
  Diffusion-Weighted Images (DWIs) are acquired with a twice-refocused spin-echo sequence, with 30 diffusion gradient directions for each of two b-values: 1000 and 2000 s/mm^2^, plus three images acquired with a b-value of 0. These parameters are optimised for estimation of the diffusion kurtosis tensor and associated scalar metrics, as well as the traditional diffusion tensor. Other parameters are: TR = 9100 milliseconds, TE = 104 milliseconds, voxel size =2 mm isotropic, FOV =192 mm × 192 mm, 66 axial slices, number of averages = 1; acquisition time of 10 minutes and 2 seconds.
4. Magnetisation Transfer Ratio (MTR) structural image
  An MTR image is constructed from two 3D, MT-prepared Spoiled Gradient (SPGR) sequences with either TR =30 milliseconds or TR =50 milliseconds (the TR =50 milliseconds sequences are used when the participant’s SAR estimation for the TR = 30 milliseconds sequences exceeds the stimulation limits); TE =5 milliseconds; flip angle =12 degree; FOV =192 mm × 192 mm; voxel-size =1.5 mm × 1.5 mm; bandwidth =190Hz/px; acquisition time of 2 minutes and 36 seconds per sequence for TR = 30 milliseconds, and 4 minutes and 19 seconds per sequence for TR = 50 milliseconds. A Gaussian shaped RF pulse with an offset frequency of 1950Hz (bandwidth =375 Hz, 500 degree flip angle, duration =9984 microseconds) is applied to one of the sequences, and the MTR calculated as MTR = (M0 - Ms)/M0, where Ms and M0 are mean signal intensities with and without the saturation pulse, respectively.
5. Resting state
  To assess intrinsic (passive) aspects of neural connectivity, T2*-weighted fMRI data are acquired while participants rest with their eyes shut using a Gradient-Echo Echo-Planar Imaging (EPI) sequence. A total of 261 volumes are acquired, each containing 32 axial slices (acquired in descending order), slice thickness of 3.7 mm with an interslice gap of 20% (for whole brain coverage including cerebellum; TR =1970 milliseconds; TE =30 milliseconds; flip angle =78 degrees; FOV =192 mm × 192 mm; voxel-size =3 mm × 3 mm × 4.44 mm) and acquisition time of 8 minutes and 40 seconds.
6. Movie watching
  To assess stimulus-driven (active) aspects of neural connectivity across a range of networks, participants watch an excerpt of a compelling but unfamiliar film. A black-and-white television drama previously used in an fMRI study [60], Alfred Hitchcock’s “Bang! You’re Dead”, was edited from a running time of 30 minutes to 8 minutes, while maintaining the plot. A total of 193 volumes are acquired using a multi-echo, T2*-weighted EPI sequence (TR =2470 milliseconds, five echoes [TE =9.4 milliseconds, 21.2 milliseconds, 33 milliseconds, 45 milliseconds, 57 milliseconds], flip angle =78 degrees, 32 axial slices of thickness of 3.7 mm with an interslice gap of 20%, FOV =192mm × 192 mm, voxel-size =3 mm × 3 mm × 4.44 mm) with an acquisition time of 8 minutes and 13 seconds.
7. Sensorimotor task
  To assess basic sensorimotor neural responses, fMRI data are acquired while participants perform a simple audio/visual sensorimotor task. In this task, participants respond to 129 trials consisting of an initial practice trial, 120 bimodal audio/visual trials, and eight unimodal trials included to discourage strategic responding to one modality (four visual only and four auditory only). The timing of trials is optimised for estimation of the fMRI impulse response by generating a sequence of stimulation and null trials using a 255-length m-sequence [61] with m = 2 and minimal stimulus onset asynchrony (SOA) of 2 seconds (resulting in SOAs ranging from 2-26 seconds). For each bimodal trial, participants see two checkerboards presented to the left and right of a central fixation (34 milliseconds duration) and simultaneously hear a 300 milliseconds binaural tone at one of three frequencies (300, 600, or 1200 Hz, equal numbers of trials pseudorandomly ordered). For unimodal trials, participants either only hear a tone or see the checkerboards. For each trial, participants respond by pressing a button with their right index finger if they hear or see any stimuli. Scanning parameters for this task are the same as in the Resting state scan.
8. Field maps
  Differences in the magnetic susceptibility of head tissues, bone and air lead to inhomogeneities in the magnetic field in the scanner, which particularly affect EPI MRI. To measure the field inhomogeneities, an SPGR gradient-echo sequence with the same parameters as the Resting state and Sensorimotor tasks are acquired, but with two TEs (5.19 milliseconds and 7.65 milliseconds). The phase difference between the two TEs can be used to calculate field maps in order to unwarp image distortions caused by field inhomogeneities. Acquisition time is 54 seconds.

#### MEG session

MEG data are acquired while participants sit within a 306-channel Vectorview system (Elekta Neuromag, Helsinki), consisting of 102 magnetometers and 204 orthogonal planar gradiometers. Data are sampled at 1kHz with a band-pass filter of 0.03-330 Hz. Head position within the MEG helmet is estimated continuously using four Head-Position Indicator (HPI) coils to allow for offline correction of head motion. Two pairs of bipolar electrodes are used to record vertical and horizontal electrooculogram (EOG) signals to monitor blinks and eye-movements, and one pair of bipolar electrodes records the electrocardiogram (ECG) signal to monitor pulse-related artefacts.

Measures gathered in the MEG session include:

1. Resting state
  To assess intrinsic aspects of neural connectivity, we gather eyes-closed resting state data from participants for approximately 8 minutes and 40 seconds, with the first 20 seconds discarded, comparable to the Stage 2 fMRI Resting state data.
2. Sensorimotor task
  To assess basic sensorimotor neural responses, MEG data are recorded while participants perform a simple audio/visual sensorimotor task identical to that in the Stage 2 MRI session. Following this, there is an additional passive stage in which 120 trials of unimodal stimuli are presented every 1 second, half with auditory tones at one of three frequencies (300, 600, or 1200 Hz) presented for 300 milliseconds, and half with a checkerboard patterns presented for 34 milliseconds, and no requirement for the participant to make motor responses. The purpose of this passive stage was to estimate auditory and visual evoked responses independently, to help separate auditory and visual responses during the main sensorimotor task.

#### Physiological measures

In order to assess core physiological factors that affect cardiovascular health, participants have height and weight measured. Height is measured with a portable stadiometer with a sliding head plate, a base plate and a connecting rod marked with a measuring scale. Weight is measured with portable battery operated electronic weighing scales.

Participants also have blood pressure measures taken. Blood pressure is measured with Digital Blood Pressure Monitor (A&D Medical UA-774). Experimenters are trained to take measurements by a trained clinician, and their measurements are recorded three times to assure reliability.

#### Saliva sample

The Cam-CAN Stage 2 participants who give specific consent are asked to deposit a small saliva sample into a collecting pot. This sample is stored for use in future genotyping analyses when appropriate supplementary funding has been secured. When looked at in conjunction with the cognitive and neural measures collected, the genotyping will help us understand the genetic influences on cognition and ageing.

### Cam-CAN Stage 3: targeted cognitive neuroscience

#### Design

Stage 3 measures include nine fMRI experiments, six MEG experiments, core MRI measures, core MEG measures, a re-assessment of cognitive health and sensory abilities, and a re-assessment of physiological measures including height, weight, and blood pressure. Materials and procedures for each task are described below. Measures are divided into four MRI sessions and two MEG sessions, and each participant attends two MRI sessions and one MEG session. The content and timing for each session are outlined in Table 4. Participants’ assigned sessions are designed to maximize the number of combinations of sessions across participants, so that different experiments can be compared across sessions and modalities. See Table 5 for a summary of how sessions are combined across participant groups. The order of sessions is not counterbalanced, but participants in Groups 1 and 4 attend fMRI Session 2 before they attend MEG Session 2. This order assures that they experience a passive version of the Sentence comprehension task (in fMRI Session 2, see description below) before providing judgments of those sentences (in fMRI Session 2 and MEG Session 2; see also Table 4).

#### fMRI sessions

fMRI sessions are conducted on the same scanner as Stage 2.

### MRI measures

In addition to Stage 3 fMRI tasks (described below), the following MR measures are acquired for each participant:

1. T1-weighted structural image
  The same parameters are used as in Stage 2 above.
2. T2-weighted FLAIR structural image
  A 2D T2-weighted Fluid Attenuated Inversion Recovery (FLAIR) image is acquired with the following parameters: TR =9000 milliseconds, TE =100 milliseconds, TI =2500 milliseconds, flip angle =150 degrees, FOV =220 mm × 220 mm × 130 mm, voxel-size =0.9 mm × 0.9 mm × 4 mm, 30% interslice gap, acquisition time of 4 minutes and 32 seconds.
3. Arterial Spin Labelling (ASL)
  In order to provide a quantitative measure of blood delivery to different brain regions, an ASL sequence is used with the following parameters: TR =2500 milliseconds, TE =13 milliseconds, TI 1 = 700 milliseconds, TI 2 = 1800 milliseconds, Saturation stop time =1600 milliseconds, flip angle =90 degrees, FOV =256 mm × 256 mm × 100 mm, voxel-size =4 mm × 4 mm × 8 mm, 25% interslice gap, acquisition time of 3 minutes and 52 seconds.
4. Resting state
  The same parameters are used as in Stage 2, except the acquisition time is slightly shorter at 5 minutes due to time constraints in Stage 3.
5. Field maps
  Because of analysis requirements of the fMRI Visual short-term memory task (see description below), in fMRI Session 4, an SPGR gradient-echo sequence is acquired with the same parameters described for the Stage 2 Field maps.

### fMRI tasks

For all but one of the fMRI tasks, the same T2*-weighted Gradient-Echo EPI sequence is used as for the Resting State and Sensorimotor sessions in Stage 2, except that the number of volumes (and hence acquisition time) depend on the length of the task detailed below. The one exception is the Visual short-term memory task (number 9 below), which uses the same multi-echo sequence that is used for the Movie watching fMRI task in Stage 2.

1. Emotional expression recognition
  In order to measure individual differences in the functional connectivity underpinning emotional responses to different facial expressions, we use an fMRI task that modulates frontal-amygdala connectivity [62,63]. This task compares brain activity when observing angry versus neutral expressions, and assesses how individuals differ in how they regulate responses to negative emotional expressions.
  Materials for this task include 30 different identities, 15 male and 15 female, each shown with one angry and one neutral expression (total 60 experimental stimuli). Participants see stimuli in 24 blocks (12 blocks of angry faces, 12 blocks of neutral faces), each 21 seconds long. Within each block, five experimental trials are presented, intermixed with five null events. For each experimental trial, a face is presented for 1000 milliseconds, followed by a fixation for 750 milliseconds, and null events are shown for 1750 milliseconds. Participants respond with a button press to each face to indicate whether the face is male or female.
  Key behavioural measures include response times in angry and neutral conditions. Neuroimaging results will not only demonstrate different patterns of amygdala activity during neutral and angry face recognition, but will measure changes in connectivity between the amygdala and prefrontal cortex when viewing angry and neutral faces.
2. Emotional memory
  This is the same task as used in Stage 2, except that the counterbalancing procedure means that only participants who do not attempt the Emotional memory task in Stage 2 are administered this task in Stage 3. Only the Study phase of the task is run in the scanner, with the Test phase conducted after participants have left the scanner (with a delay of approximately 10 minutes, as in Stage 2). This means that encoding-related brain activity during Study can be related to whether a stimulus is or is not correctly remembered later in Test (so-called “subsequent memory effects”). This brain activity can be further distinguished according to the emotional valence of the stimulus (positive, neutral or negative), and whether memory was accompanied by priming, familiarity and/or recollection. We expect that the different memory measures will be differentially related to age, and related to partially-dissociable neural systems.
3. Emotional reactivity and regulation
  This task is very similar to that used in Stage 2, except that the counterbalancing procedure means that only participants who do not attempt the Emotional reactivity and regulation task in Stage 2 are administered this task in Stage 3. Trials follow a similar procedure as the Stage 2 task, so that for each trial, participants receive a prompt to indicate the valence and how they should observe the film (e.g. “WATCH NEUTRAL” or “REAPPRAISE NEGATIVE”). This is followed by a 30 second film clip after which participants rate their emotional response on a single scale ranging from “very negative” to “very positive”. Finally, after viewing a positive or negative video, participants see a calming “washout” clip before the next trial. Key behavioural measures are similar to those in the Stage 2 task, including emotional response ratings for all experimental conditions and the comparison of watch and reappraise conditions. Neuroimaging analyses likewise will include the comparison of brain activity during different conditions, as well as an assessment of neural connectivity during watch and reappraise conditions.
4. Fluid intelligence
  This task provides a measure of neural activity underpinning core fluid intelligence processes [54]. It involves a fluid intelligence task modified for use in the scanner [64], which is based on the classification subtest in the standardized Cattell Culture Fair test of fluid intelligence (see Stage 2 Fluid intelligence task).
  The task consists of a series of puzzles where participants are presented with a display of four patterns. Their task is to select the “odd one out” by using a button press to select one of the four patterns. The basis for a correct decision is sometimes relatively easy, such as a gross difference in shape or pattern, and it is sometimes relatively difficult, requiring the identification of abstract patterns to detect the “odd one out”.
  The task in the scanner employs a block design, where participants solve alternating blocks of easy and difficult trials, lasting 30 seconds each. In total, participants complete four blocks of easy and four blocks of difficult problems. On each trial a stimulus appears and remains on the screen until the participant responds, with the block automatically ending after 30 seconds and the next block beginning immediately. Participants are encouraged to puzzle over each trial for as long as necessary, only responding when they are confident of the correct answer. This design means that the number of trials in a block varies across individuals, but the time spent on each type of problem (easy and difficult) is held constant.
5. Free selection
  This task is a visually paced right hand button press task used previously to study executive control and action decisions in ageing and neurodegenerative disease [65–67]. Participants are presented with an image of a right hand and press a button with one of their four right hand fingers in response to a cue. The cue is either a “specified” cue in which a single opaque circle indicates which finger to press, or “chosen” cue in which all circles appeared opaque indicating participants must choose a finger to press. In both cases, participants are instructed to respond as quickly as possible. The task includes 40 specified trials (10 for each finger) and 40 action selection trials, interspersed with 40 null events in which no cue is presented. Cues are presented for 1 second with a stimulus onset asynchrony of 2.5 seconds, and are pseudorandomly ordered so that participants do not see four or more responses of the same condition (action selection, specified or null) in a row.
6. Picture naming
  This task taps into the neural systems that underpin lexical retrieval during picture naming, including semantic access and mapping semantic to phonological representations. Normal ageing is associated with increased word finding failures, and this object naming task will help identify the neural dynamics associated with successful and failed object naming. This task uses the same materials and procedure as the baseline phase in the Stage 2 Picture-picture priming task. Participants are scanned while naming pictures, but there is no priming manipulation and no systematic phonological or semantic relatedness between sequential objects.
  Participants name aloud a series of 200 pictures of common objects with short (one or two syllable) names, presented in a different random order for each participant. On each trial, a fixation point is presented for 500 milliseconds, followed by an object for 750 milliseconds, followed by a blank screen for 1000 milliseconds. In addition to experimental trials, participants see two low level visual baseline conditions: 30 trials of phase-scrambled images of target objects, and 30 fixation crosses. Participants respond “noise” to the scrambled images and make no response to the fixation crosses. Participants are instructed to name every picture as quickly and accurately as possible. Their responses are recorded to a digital sound file as well as scored for accuracy by an experimenter.
  Key behavioural measures are similar to those of the Stage 2 task, including naming speed and accuracy. Key contrasts in the neuroimaging analyses include identifying neural systems involved in naming meaningful versus scrambled objects and successful versus failed naming attempts.
7. Sentence comprehension
  The aim of this experiment is to investigate syntactic processing in on-line comprehension of spoken sentences, and uses the design, materials and procedure included in the “syntax” conditions of the Stage 2 sentence comprehension task. Previous research suggests age-related preservation of core aspects of language comprehension, which are largely underpinned by a left-lateralised fronto-temporal network. However, most experiments evaluating language comprehension involve tasks with components that can be affected by age, including speeded responses, decision making, divided attention, or memory loads. This experiment aims to examine the neural systems underpinning syntactic processing and how these interact with other networks related to cognitive demands.
  This experiment involves two phases, a Natural Listening phase and a Task phase. Both phases include sentences from the materials used in the Stage 2 sentence comprehension task, including 42 unambiguous sentences and 84 sentences containing syntactically-ambiguous phrases, 42 continuing with the dominant interpretation and 42 continuing with the subordinate interpretation. In addition to these sentences, materials included 21 auditory baseline stimuli which consisted of envelope-shaped ‘musical rain’ [68] in which the long-term spectrotemporal distribution of energy is matched to that of the corresponding speech stimuli.
  In the Natural Listening phase, participants listen to full versions of randomly ordered stimuli, spoken in a female voice, and make no overt response. In the Task phase, the procedure is identical to that of the Stage 2 sentence comprehension task: participants hear sentences up to and including the ambiguous/unambiguous phrase and after a 200 milliseconds delay hear a continuation word in a male voice. They make button-press responses to indicate whether the continuation is acceptable or unacceptable, and make no responses to ‘musical rain’ trials.
  Key behavioural measures from the Task phase are the same as in the Stage 2 task: comparison of “unacceptable” judgment rates and response times in the unambiguous, dominant, and subordinate conditions. Likewise, key neuroimaging analyses involve determining the neural systems active during comprehension of unambiguous, dominant, and subordinate conditions, and the comparison of Natural Listening and Task phases. Of particular interest is evaluating how ambiguity and task requirements affect connectivity within the language system and between the language system and other cognitive systems.
8. Stop-Signal, Go/No-Go
  This task assesses systems involved in action restraint and action cancellation by randomly interleaving trials that are typical of so-called “Stop-Signal” tasks with those of so-called “Go/No-Go” tasks [69,70], so that trials are either “Go” (360 trials), “No-Go” (40 trials), or “Stop-Signal” (80 trials). On Go trials, participants view a black arrow pointing left or right (duration 1000 milliseconds) and indicate the direction of the arrow by pressing left/right buttons with their right hand. On Stop-Signal trials, the black arrow changed colour (from black to red) concurrent with a tone, after a short variable Stop-Signal delay. Participants are instructed that they should not respond to the red arrow, so stop signal trials require cancelling their initial response to the black arrow. The Stop-Signal delay varies from trial to trial in steps of 50 milliseconds, and is titrated to participants’ performance using a tracking algorithm to maintain 50% successful inhibition. Finally, in No-Go trials, participants are required to make no response to a red left/right arrow (duration 1000 milliseconds) and concurrent tone, equivalent to a Stop-Signal delay of zero. These trials require participants to restrain the response to the arrow. Four key parameters of interest are measured: the rate of Go commission errors (left/right response is incorrect), mean reaction time of correct Go trials, rate of No-Go commission errors, and an estimate of the time needed to inhibit responses on the Stop-Signal trials. This “Stop-Signal Response time” is estimated by subtracting mean Stop-Signal delay from ‘finishing time’ of the stop process using the integration method [71], and corrected for the Go omission rate (see [69]).
9. Visual short-term memory
  This task examines the neural systems underpinning VSTM, and has key features in common with the VSTM task used in Stage 2, including a continuous report of a probed feature from memory.
  Materials and procedure are closely based on a task used by Emrich et al. [56]. On each trial, participants see three arrays of coloured dots, one red, one yellow, and one blue. The dot displays are presented in quick succession: a 250 milliseconds fixation is followed by a 500 milliseconds dot display. As a manipulation of set size, one, two, or three of the dot displays move in a single direction which must be remembered. The other displays rotate around a central axis, and these rotating distractor displays can be ignored. After the third display, there is an 8 second delay, followed by the probe display. The probe display has a coloured circle to indicate which dot display to recall (red, yellow, or blue). The circle contains a pointer that can be adjusted to indicate which direction the target dot display had been moving. Participants have 5 seconds to adjust the pointer to match the direction of the to-be-remembered dot display. On 90% of trials the probed movements are in one of three directions (7, 127, or 247 degrees).
  As with the Stage 2 VSTM task, for each set-size, behavioural measures of VSTM quantity and quality are estimated by fitting the error distribution with a mixture model approach developed by Zhang and Luck [57]. Key neuroimaging analyses will relate performance measures to activity in networks associated with visual processing and short term memory. Additionally, we will employ a Multi-Voxel Pattern Analysis (MVPA) approach to evaluate the contents of short term memory by classifying the dot displays by their direction of movement (7, 127, or 247 degrees). The success of this classification will be related to memory load (set size) and individual differences in performance [56].

#### MEG sessions

MEG data are acquired in the same fashion as in Stage 2.

### MEG measures

1. Resting state
  The same parameters are used as in Stage 2, except the acquisition time is slightly shorter at 5 minutes due to time constraints in Stage 3.

### MEG tasks

1. Incidental memory
  This experiment assesses the degree to which the brain automatically detects repetition of a stimulus, even when the participant is not trying to remember the stimulus (i.e. repetition is incidental to the main task, which is to detect a rare target). Previous research has shown that evoked fields differ between initial and repeat presentations from 300 to 500 milliseconds after the stimulus onset over anterior temporal sites.
  The task involves viewing complex scenes (e.g. building interiors, landscapes, or cityscapes), and responding to scenes which contain a moon. Scenes are randomly intermixed with the constraint that each scene repeats after 14-93 (median =42) intervening stimuli. There are 562 trials in total, consisting of 24 “burn-in” trials followed by 256 occurrences of both initial and repeat presentations of each scene repetitions, plus 26 target pictures (those that contain a moon).
  Participants are told to press a key each time they see a moon, and trials containing a target or other trials with a key press are discarded prior to analysis. Each trial starts with a fixation cross that is presented for between 100 and 300 milliseconds (average 200 milliseconds), followed by a scene presented for 800 milliseconds, with the experiment lasting approximately 9.5 minutes in total. Key neuroimaging measures for this task are differences in evoked fields and induced energy for initial relative to repeat presentations.
2. Multi-mismatch
  This task measures neural responses to unpredictable auditory events, which have been shown to depend on fronto-temporal interaction, and is impaired in dementia [72,73]. The task is adapted from the multi-feature ‘Optimum-1’ paradigm [74]. The stimuli comprise a block of harmonic tones presented every 500 milliseconds in three blocks of 5 minutes each while participants view a silent natural history film. The standard tone has a duration of 75 milliseconds and contains three sinusoidal partials of 500, 1000 and 1500 Hz. The five deviant tones differ from the standard by either frequency band (550, 1100, and 1650 Hz), intensity (+/- 6Db), duration (25 vs. 75 milliseconds), side of sound source (left or right rather than bilateral), or by a silent gap (silent gap of 25 milliseconds in the middle). A block starts with fifteen standard tones, after which standard tones are alternated with deviant tones. The order of deviant tones is permuted, such that in a sequence of 10 tones, each deviant is presented once and the same deviant type is never immediately repeated. There are a total of 900 standards and 900 deviants. Each participant’s hearing is checked before the beginning of the task to assure tones are audible, and tones are presented binaurally via plastic tubes and earpieces. The task is passive, with no behavioural measures.
3. Picture naming
  Like the fMRI Picture naming task, this task taps into the neural systems that underpin lexical retrieval, including conceptual access and mapping semantic to phonological representations. Using MEG enables us to examine the temporal dynamics of conceptual and phonological access during successful and erroneous naming. In particular, the temporal resolution of MEG provides the ability to separate the temporal aspects of semantic activation, phonological access, and the recruitment of domain-general processes that are involved in overcoming naming difficulties.
  Materials and procedure are closely modelled on those of Clarke, Taylor, Devereux, and Tyler [75]. Participants name aloud 302 coloured pictures of objects. On each trial, a fixation point is presented for 500 milliseconds, followed by an object for 500 milliseconds, followed by a blank screen for 2400-2700 milliseconds. Participants name every picture as quickly and accurately as possible. Their responses are recorded to a digital sound file and scored for accuracy by an experimenter.
  Key behavioural measures are similar to those of the fMRI task, including naming speed and accuracy. Key neuroimaging measures include the time courses of successful versus failed naming attempts.
4. Sentence comprehension
  This task exposes the dynamics of how neural activity is modulated by syntactic processing during sentence processing. Previous research suggests that syntactic analysis of spoken utterances involves the co-activation of left inferior frontal regions (typically Brodmann Areas 45 and 47) and left posterior middle temporal regions (e.g. [76–78]). This task examines the timecourse of activation and connectivity across the brain as a function of syntactic processing.
  The materials and procedure are similar to those in the Task phase of the fMRI sentence comprehension experiment, and the “syntax” conditions of the Stage 2 Sentence comprehension task. Materials include 66 unambiguous sentences and 132 ambiguous sentences, 66 resolving to the dominant interpretation and 66 resolving to the subordinate interpretation. On each trial, participants hear sentences up to and including the ambiguous phrase (or matched phrase in unambiguous sentences), and after a 200 millisecond delay hear a continuation word in a male voice. They make button-press responses to indicate whether the continuation is acceptable or unacceptable.
  Key behavioural measures from the Task phase are the same as in the Stage 2 Sentence comprehension task: comparison of rejection rates and response times in the unambiguous, dominant, and subordinate conditions. MEG analyses will examine the timing of the activation and resolution of syntactic ambiguity and their relationship to neural oscillations across the life-span. We will also use Representational Similarity Analysis (RSA) to tap into linguistic representations over time [79] to determine how they change with age.
5. Stop-Signal, Go/No-Go
  The Stop-Signal, Go/No-Go task for MEG is the same as the fMRI version of the task (see above), except that it is administered in three blocks of 8 minutes, with a total of 660 trials: 480 Go trials, 60 No-Go trials and 120 Stop-Signal trials. Principle behavioural outcome measures are the same as for the fMRI task.
6. Word recognition
  This task assesses basic properties of visual word recognition, in particular the process that maps visual inputs onto lexical form and meaning. Initial stages of this mapping involve analysis of visual form and orthography which engages occipito-temporal cortex, most strongly on the left, and that later stages of lexical access and interpretation involve middle temporal and fronto-temporal regions, also primarily in the left hemisphere.
  Early stages of recognition are dominated by an automatic decomposition of the word into morphemic units, for example segmenting the complex word *hunter* into the root word *hunt* and the suffix –*er*. This early processing is strongly bottom-up, and blind to lexical constraints. For example, the same segmenting occurs for “pseudo-complex” words like *corner*, despite the fact that in this case the root word *corn* is not related to the meaning of *corner*. After segmentation, these pseudo-complex words require additional processing compared to real complex words in order to access the correct meaning.
  In this experiment, stimuli vary in the presence or absence of a root word and a suffix, and whether the combination of the root and suffix results in a meaningful word. There are six main conditions: (1) Real complex words like farmer have both a root and a suffix, and the meaning is related to the root word (e.g. farm); (2) Pseudo complex words like corner have both a root word and a suffix, but the meaning is not related to the root (e.g. corn); (3) Non-words contain a root and suffix but do not form a valid English verb when combined (e.g. goated). Two further conditions include (4) words with a root but no suffix (e.g. scandal) and (5) words with neither a suffix nor root word (e.g. biscuit). Finally, (6) consonant strings matched in length to the word conditions are included to examine early visual word processing.
  In the experiment, participants passively read 50 written letter strings in each of the six word conditions, plus 80 length-matched consonant strings, which are presented in random order. Each of 380 experimental trials consists of a fixation cross which appears for 500 milliseconds, and is followed by the stimulus for 200 milliseconds.

#### Physiological measures retest

Participants have height, weight, and blood pressure measures from Stage 2 repeated. Experimenters use the same equipment and procedures as in Stage 2, which occur in the same setting.

#### Cognitive assessment retest

Participants have the Mini Mental State Exam (MMSE) from Stage 1 repeated. Experimenters use the same computer programme to administer the test as is used in Stage 1.

#### Sensory assessment retest

Participants have the hearing and vision checks from Stage 1 repeated. Experimenters use the same equipment and procedures as are used in Stage 1.

## Discussion

Life expectancy in the UK has increased by over 30 years in the last century. This reflects a wider international trend with major implications for the development of economic, social and health policy at local, national, and international levels. Cognitive change through the healthy lifespan is a topic of urgent scientific and social concern. The Cam-CAN programme will establish a large population-based representative participant group to satisfy multiple levels of investigation. The study results will be used both nationally and regionally for health policy and future health planning. We will use the data to understand the effects of ageing both within and across the major cognitive domains, by examining the relationship between neural structure, neural function, and cognitive performance. This will allow us to define the neural networks supporting specific cognitive domains and identify selective vulnerabilities of different brain networks to the effects of ageing. Our detailed analysis of neural and cognitive flexibility will help us to identify what characterizes older adults with preserved performance, a perspective with huge implications for how society views the ageing process. Moreover, we will be able to identify the conditions underpinning successful cognitive ageing, in particular the factors associated with neural flexibility. By using a population-based representative cohort of such a size, we can ensure that the observed patterns of neural change are relevant to the population at large. Finally, because our findings will help specify normal age-related deficits, they will show how normal ageing differs from pathological ageing in conditions such as Alzheimer’s disease.
